# Non-invasive assessment of hormonal fluctuations during pregnancy in guanacos (*Lama guanicoe*) and its application in a wild population

**DOI:** 10.1093/conphys/coae003

**Published:** 2024-02-09

**Authors:** A Marozzi, V I Cantarelli, A Panebianco, F M Gomez, R Ovejero, P F Gregorio, F Peña, M F Ponzio, P D Carmanchahi

**Affiliations:** Grupo de Investigaciones en Ecofisiología de Fauna Silvestre, Instituto de Investigaciones en Biodiversidad y Medioambiente, Consejo Nacional de Investigaciones Científicas y Técnicas (CONICET) - Universidad Nacional del Comahue (UNCo), 235 Pasaje de la Paz St., (8370) San Martín de los Andes, Argentina; Instituto de Investigaciones en Ciencias de la Salud (CONICET – INICSA), Facultad de Ciencias Médicas, UNC, De la Reforma Bv. and Enfermera Gordillo St., Pabellón de Biología Celular. Ciudad Universitaria, (5016) Córdoba, Argentina; Grupo de Investigaciones en Ecofisiología de Fauna Silvestre, Instituto de Investigaciones en Biodiversidad y Medioambiente, Consejo Nacional de Investigaciones Científicas y Técnicas (CONICET) - Universidad Nacional del Comahue (UNCo), 235 Pasaje de la Paz St., (8370) San Martín de los Andes, Argentina; Asentamiento Universitario San Martín de los Andes, CONICET, UNCo, 235 Pasaje de la Paz St., (8370) San Martín de los Andes, Argentina; Instituto de Ecología Regional (CONICET - Universidad Nacional de Tucumán), Residencia Universitaria Horco Molle, Edificio las Cúpulas (4107) Yerba Buena, Argentina; Grupo de Investigaciones en Ecofisiología de Fauna Silvestre, Instituto de Investigaciones en Biodiversidad y Medioambiente, Consejo Nacional de Investigaciones Científicas y Técnicas (CONICET) - Universidad Nacional del Comahue (UNCo), 235 Pasaje de la Paz St., (8370) San Martín de los Andes, Argentina; Witral, Red de Investigaciones en conservación y manejo de vida silvestre en sistemas socio-ecológicos, Instituto Argentino de Investigaciones de Zonas Áridas, CONICET,Av. Ruiz Leal s/n, Parque General San Martín (5500) Mendoza, Argentina; Asentamiento Universitario San Martín de los Andes, CONICET, UNCo, 235 Pasaje de la Paz St., (8370) San Martín de los Andes, Argentina; Grupo de Investigaciones en Ecofisiología de Fauna Silvestre, Instituto de Investigaciones en Biodiversidad y Medioambiente, Consejo Nacional de Investigaciones Científicas y Técnicas (CONICET) - Universidad Nacional del Comahue (UNCo), 235 Pasaje de la Paz St., (8370) San Martín de los Andes, Argentina

**Keywords:** Estrone conjugates, gestation, pregnancy rate, pregnanediol glucuronides

## Abstract

Obtaining endocrinological profiles using non-invasive methodologies by the measurement of hormone fecal metabolites is a widely used method to monitor ovarian activity and pregnancy in wild species. These tools allow the obtention of physiological information without causing capture-related stress on the individuals. In this research, we aimed to 1) biologically validate a non-invasive method to assess fecal progestagens and estrogens fluctuations during gestation in guanacos (*Lama guanicoe*) and 2) apply this technique to assess pregnancy in a wild free-ranging population. Fecal samples were collected through the gestation period (~12 months) of female guanacos in a 6.5-ha paddock. An increase in fecal metabolites of both hormones was detected. Progestagens increased gradually, in contrast to estrogens, which remained at basal values for most of the gestation period and peaked only a few days before calving. To assess pregnancy in wild free-ranging animals, fecal samples were collected from a population of La Payunia provincial reserve (Mendoza, Argentina) during the beginning of gestation and at the end of gestation. Through the first months of possible gestation, pregnant females represented between 40 and 80% of the population; at the end of gestation, only 20–40% of the females had confirmed pregnancies. Our results demonstrated that the polyclonal antisera and sexual hormone metabolite assays used here detect variations in the metabolites excreted through feces in guanacos and provide the possibility of non-invasive hormone monitoring of female reproductive status. Also, the findings in wild conditions suggest that natural abortions could have occurred during the first months of gestation. Although some abortions may be natural, the harsh environmental conditions that challenge the support of such a long gestational process may be another relevant factor to consider. The results obtained here enhance our understanding of the reproductive physiology of one of the most emblematic ungulates in South America.

## Introduction

Steroid hormones play a fundamental role in the reproductive physiology of females. These hormones participate in gamete production and the maintenance of pregnancy (Christensen *et al*., 2012). As a consequence, the proportion of pregnant females can be considered a starting point for assessing vital parameters like birth or recruitment rate (the process of adding new individuals to a population through reproduction or immigration; Gaillard *et al*., 2008) because individuals must reproduce to sustain population dynamics (Cain *et al*., 2012; Decesare *et al*., 2012; Kersey and Dehnhard, 2014). Gestation success can be considered an indicator of population health (Lasley and Kirkpatrick, 1991; Kirkpatrick *et al*., 1993), given that if a population is in a challenging environment, resource allocation to reproduction may not be an obvious choice, and most individuals might favor survival over reproduction (Piasecke *et al*., 2009; Nystrand and Dowling, 2020). In this sense, monitoring ovarian activity is one of the first actions we should consider to infer pregnancy rates and gestation success in wild populations (Hodges *et al*., 2010).

Obtaining endocrinological profiles using a non-invasive methodology is one of the most widely used tools to monitor ovarian activity and pregnancy in wild species (Sontakke, 2018). These techniques allow stress-free sampling with no need to capture and extract blood (Kirkpatrick *et al*., 1993; Schwarzenberger *et al*., 1995; Schwarzenberger, 2007; Mastromonaco *et al*., 2015; Flacke *et al*., 2017; Valenzuela-Molina *et al*., 2018; Miller *et al*., 2021; Watson *et al*., 2023). They rely on the fact that blood-circulating hormones are metabolized in the liver and excreted in the feces. As a result, the variation in hormone levels can be estimated using fecal metabolite dosages (Palme *et al*., 2005; Schwarzenberger and Brown, 2013). In general, the excretion rate is proportional to the amount of circulating hormone; therefore, the values obtained reflect individual endocrinological variations (Kersey and Dehnhard, 2014). Nevertheless, because secretory profiles differ across species, it is critical to demonstrate that hormonal fluctuations in the ovary are reflected in fecal metabolite concentrations via biological validation of the method in the study model (Palme *et al*., 2005).

The guanaco (*Lama guanicoe*) is the most important native herbivore in the Patagonian steppe (Carmanchahi *et al*., 2022); however, several facts about its reproductive physiology remain unclear. This species is known to be an induced ovulator (Fowler and Bravo, 2010; Riveros *et al*., 2010). Estrogens vary their concentrations according to follicular recruitment, but in the absence of mating, there is no ovulation or luteal phase. Progestagens only increase if females become pregnant; otherwise, they remain basal (Bravo *et al*., 1990; Miragaya *et al*., 2004; Riveros *et al*., 2009, 2010). In wild conditions, reproduction only occurs in austral, late spring and early summer, i.e., December, possibly due to environmental factors such as nutrient availability (Sumar, 1994; Urivola García and Riveros, 2017), photoperiod (Urivola García and Riveros, 2017; Correa *et al*., 2020), climate conditions and migratory movements (Candino *et al*., 2022).

Gestation in the guanaco lasts almost a year, between 335 and 360 days (Riveros *et al*., 2010). Studies performed on serum samples obtained every 15 days indicated that progesterone levels reach their maximum values between 260 and 290 days of gestation. After that, progestagens decrease, returning to baseline levels after calving. On the other hand, estradiol increases from day 290, reaching its maximum levels in the postpartum period (Vaughan and Tibary, 2006; Riveros *et al*., 2009). Competent dominant follicles are rapidly developed after calving to be ovulated during the early post-calving period (Riveros *et al*., 2015).

Although understanding gestation success in wild populations can provide insight into population health and dynamics, a comprehensive study of the ovarian activity using non-invasive methods has never been conducted in guanacos. To successfully study wild populations, it is necessary to develop protocols that allow the sampling of wild individuals while avoiding or minimizing human contact. In this study, we aimed to 1) biologically validate a non-invasive method to assess sexual steroid hormonal changes during gestation in guanacos kept in captivity through fecal progestogens and estrogens metabolites quantification and 2) apply this non-invasive method to diagnose early and late pregnancy stages in a wild guanaco population.

## Materials and Methods

### Ethical Statement

The experimental methodology described here was evaluated and approved by the CICUAL (Institutional Committee for the Care and Use of Laboratory or Experimental Animals) of INIBIOMA-CONICET-UNCo, Argentina, under protocol N° 2020–021. The research was also approved by the Secretary of Territorial Development and Environment (Disp. 002/20) of Neuquén Province (Argentina).

**Fig. 1:**
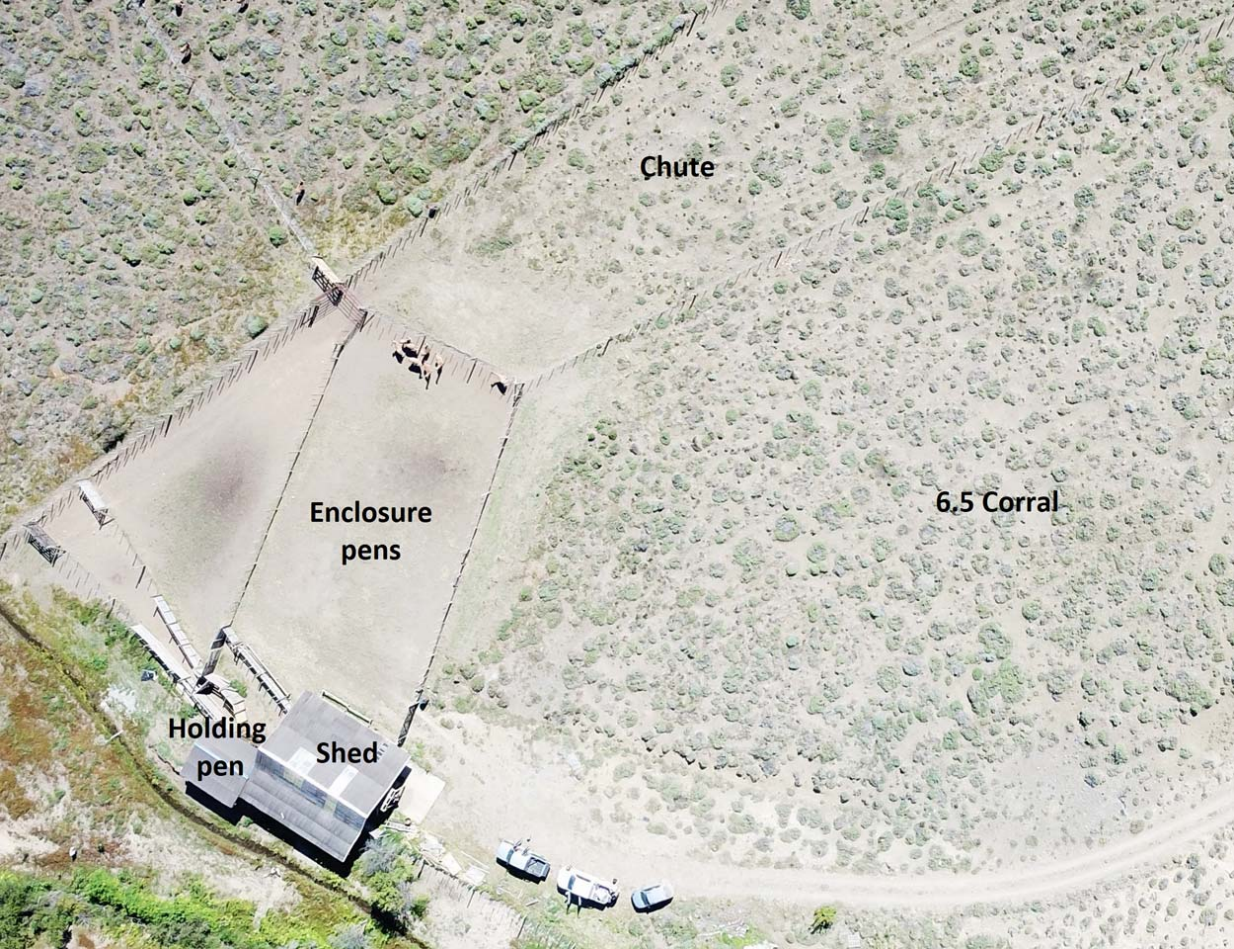
Structure used for animal handling. 6.5-ha paddock with natural pasture, free water and shelter with a chute that leads to pens for enclosing the animals. These enclosure pens open up to a holding pen that connects to a shed for handling the guanacos.

**Table 1:** Females’ ID, birth date, age and body condition score at the beginning of the experiment

**ID**	**Females’ birth date (mm/dd/yyy)**	**Age in months**	**Body condition score**
I-432	01/26/2009	107	2.5
I-436	01/28/2009	107	3
K-504	12/22/2010	84	3
K-514	12/17/2010	84	3
K-550	01/12/2011	83	3.5
K-568	01/15/2011	83	3.5
K-592	01/26/2011	83	3

### Study under captive conditions

This study was performed at ‘Los Peucos’ ranch (39°43´40.12” S; 71°03′37.58” W; Neuquén Province, Argentina). The site holds a herd of 400 guanacos in extensive farming, maintained for fiber production. To fulfill the first objective, during late spring (November 2018), seven female guanacos and one male were placed in a 6.5-ha paddock with access to natural pastures, water and shelter (Fig. 1). This time of year matches the beginning of the reproductive season in wild conditions (Franklin, 1983; Young and Franklin, 2004). Each female was identified with a different colored collar to facilitate recognition from a distance. In addition, the ranch keeps individuals marked with a tag containing a combination of numbers and letters, allowing us to know their age. We selected middle-aged females between 7 and 9 years old (Table 1). We assessed body condition by palpating the degree of sharpness of spinous processes, muscle mass and fat cover adjacent to the lumbar vertebrae (Audige *et al*., 1998; Taraborelli *et al*., 2017). Scores range from 1 (thin) to 5 (obese) (Table 1). The females remained in the paddock with the male until late March 2019, after which it was removed. Abdominal ultrasound scans were performed on the females using a scanner (SonoScape A5; SonoScape Medical Corp.) with a multifrequency probe (3–7 MHz) to confirm pregnancy in March. Additionally, we did a monitoring ultrasound in November 2019. Fecal samples were collected from all pregnant females once every 20 days from December 2018 until the last calving in February 2020.

For sample collection, each female was followed around the paddock at a distance of ~100 m and monitored with binoculars and telescopes to collect the samples immediately after defecation. Since the beginning of the birth season, i.e. December, we monitored the individuals every day between 8:00 and 18:00 Hs. to ensure that the collection of postpartum fecal samples began immediately after calving. Postpartum samples were collected every day for 6 days. All samples were placed in individual plastic bags, stored in a cooler with refrigerant gels during the day and then in a freezer at −20°C until analysis.

To estimate the day of conception, a mean gestation time of 347 days was assumed (Fowler and Bravo, 2010). We recorded the calving date and, considering it zero counted 347 backwards as the date of conception (Table 2). Of the seven pregnant females, confirmed by ultrasound at the beginning of the experiment, five gave birth to a living calf, while two had an abortion.

**Table 2:** Estimated conception date of each female in the study

**ID**	**Calving date**	**Estimated conception date**
I-432	12/09/2019	27/12/2018
I-436	12/18/2019	5/1/2018
K-504	12/13/2019	31/12/2018
K-514	Abortion	Undetermined
K-550	Abortion	Undetermined
K-568	2/1/2020	19/2/2019
K-592	1/1/2020	19/1/2019

**Table 3:** Summary of the analysed periods according to gestation time; calving day is considered as day zero; gestation days are counted in negative numbers and postpartum days, with positive numbers. The beginning of gestation is considered in the interval between (−320, −240) days and the end of gestation corresponds to the interval between (−60, 0) days

Stage of gestation	Days until/after calving
Beginning	(−347, −240)
Early	(−220, −160)
Middle	(−140, −80)
Advanced	(−60, 0)
Postpartum	(1, 6)

**Fig. 2:**
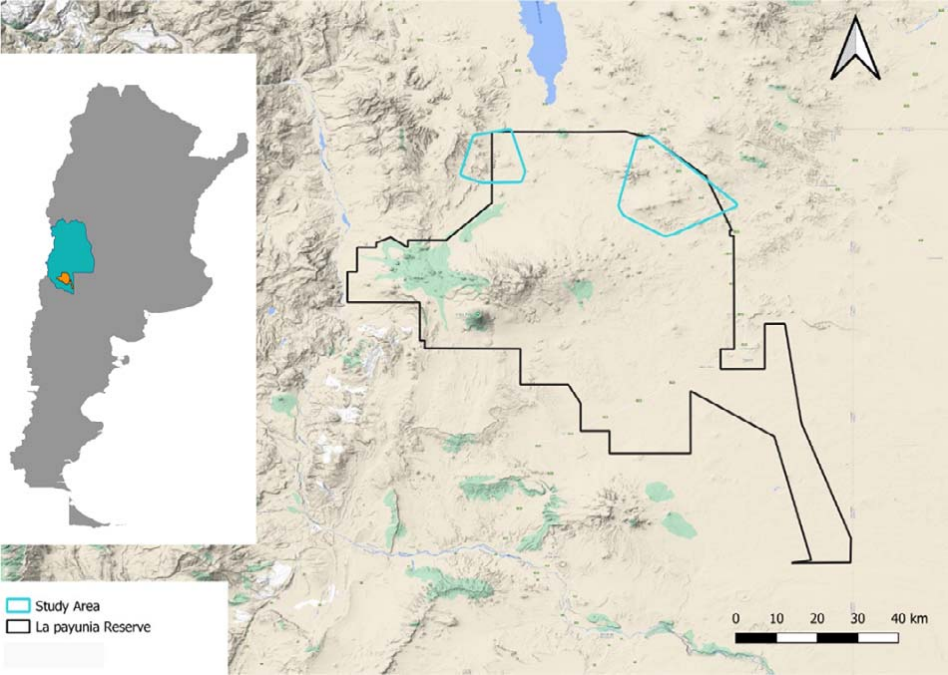
Limits of La Payunia Reserve (Mendoza, Argentina). The small polygons in the north represent the areas under study.

**Table 4:** Fieldwork summary conducted at La Payunia and the number of samples taken in each survey

Year	Month of collection	Number of samples (*n*)
2007	February–March	9
2007	September–October–November	32
2008	April	16
2008	October	18
2016	September–October	63
2017	February	44
2017	September	31
2018	February	31

For analysis of hormones fecal metabolites data during gestation, a mixed effect model was used with time as a fixed effect and females as a random effect with the MCMCglmm package of R (Hadfield, 2021). A normal prior distribution was established for the random variable. The hormonal data was re-grouped into four periods: beginning of gestation (the first 107 days), early gestation (next 60 days), mid-gestation (next 60 days) and late gestation (the last 60 days; Table 3).

Data on pre-calving and post-calving hormonal variations were separately analysed due to differences in sampling frequency (once every 20 days approximately for pre-calving samples and once a day for post-calving samples). In the case of pre-calving, we considered advanced gestation (between 0 and 60 days) as the reference level. In the case of postpartum samples, day zero (calving day) was taken as the reference level (Hadfield, 2021). We considered significant differences among stages of gestation if credible intervals did not overlap zero. Also, the effective number of Markov chains (n_eff_) was assessed.

### Study in wild conditions

To evaluate the gestation rate success in the wild, we worked with a guanaco population at La Payunia Provincial Reserve (Mendoza, Argentina; 36°25′S; 69°12′W) with an area of 6.641 km^2^. This protected area presents a transitional environment between the Patagonian steppe and the Monte (Martínez Carretero, 2004) and has one of the most important wild partially migratory guanaco populations in South America, estimated at 26 000 individuals (Schroeder *et al*., 2013). The north of the reserve (Fig. 2) is the preferred area of the population during the breeding season from September to March (Bolgeri, 2016). The sampling designed for this research consisted of traveling along the available roads of the north of the reserve to collect fecal samples of female guanacos randomly.

**Fig. 3:**
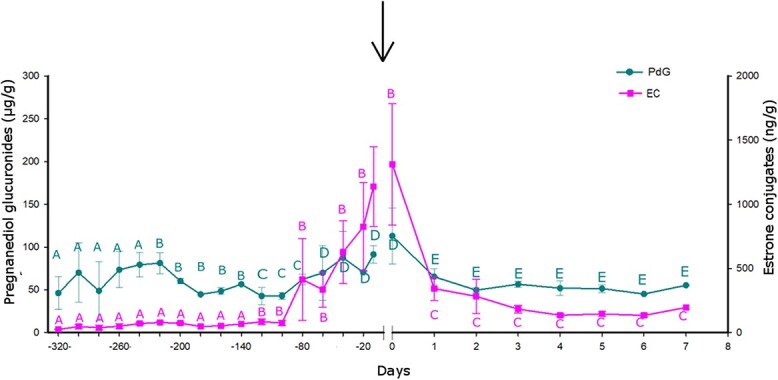
Average PdG and EC concentrations in pregnant females from Los Peucos ranch. The arrow indicates the time of birth. Different letters indicate that credible intervals do not overlap zero; consequently, there are meaningful differences in fecal metabolite concentrations. The same letter indicates no meaningful differences among concentrations.

Fecal samples were collected from female guanacos at the beginning of pregnancy (February, March and April, Table 4) and in their final stages (September, October and November, Table 4). The observers moved through the roads inside the reserve at low speed in a pickup truck; when a group of guanacos was spotted, it was observed using binoculars and a telescope. When an individual defecated, we assigned sex and collected the samples from females, stored them in a plastic bag and kept them in liquid nitrogen (−196°C) before arrival at the laboratory, where samples were stored in a freezer at −20°C until analysis. We used samples collected in 2007, 2008, 2016, 2017 and 2018 (Table 4). We estimated the percentage of pregnant and non-pregnant females in the periods studied. To assess differences in the proportion of these two groups among years, we did a proportion test using R (R Core Team, 2020).

### Laboratory analysis

The endocrine patterns of dams under captive and wild conditions were estimated through the analysis of fecal estrone conjugates (EC) and pregnanediol glucuronides (PdG) concentrations determined with an in-house enzyme immunoassay (EIA) using polyclonal antibodies. Standards and their corresponding horseradish peroxidase conjugates were used (anti-EC R522–2 and anti-PdG R13904; CJ Munro, UC Davis, CA, USA) as previously described by Marozzi *et al*. (2020). Before the assay, and according to parallelism results, fecal extracts were diluted in EIA buffer (0.1 mM 165 sodium phosphate buffer, pH 7.0, containing 9 g of NaCl and 1 g of BSA per litre; final dilution: EC 1:100, PdG 1:20) and assayed in duplicate.

Cross-reactivity reported for EC is as follows: estrone 3-glucuronide 100%; estrone 3-sulfate 66.6%; estrone 23.8%; estradiol 17b 7.8%; estradiol 3-glucuronide 3.8%; estradiol 3-sulfate 3.3%; estradiol 17-sulfate 0.1%; estradiol 3-disulfate 0.1%; and <0.1 with all other steroids tested. Cross-reactivity reported for PdG is as follows: pregnanediol 3-glucuronide 100%; 20a-Hydroxy progesterone 44.8%; 20b-Hydroxy-progesterone 3.1%; progesterone 0.7%; estradiol 17b 0.04%; testosterone 0.2% and cortisol 0.06%. The assay sensitivities for EC and PdG were 0.0078 and 1.954 ng/ml, respectively. The intra-assay coefficient of variation was <12% for both hormones; the inter-assays were 6.5% for EC and 6.8% for PdG.

## Results

### Study under captive conditions

The calving season started in December (date of first calving, December 9, 2019) and ended in February (date of last calving, February 1, 2020, see Table 2). During gestation, we detected an increase in fecal metabolites in both hormones. Progestagen metabolites showed slight fluctuations through pregnancy, increased gradually from the beginning of gestation (days −347, −240) and immediately decreased to baseline levels after calving (Fig. 3). On the contrary, estrogens persisted at basal values throughout pregnancy and increased abruptly in the last 60 days before calving (Fig. 3; Table 5). Already on postpartum day 1, a sharp decrease in estrogen metabolite concentrations was detected (Fig. 3; Table 6). When concentrations were considered in periods (Table 3), prepartum PdG concentrations were significantly lower between the early and middle gestation compared to the end of gestation (Table 7). Similar to EC, PdG concentrations decreased on postpartum day 1 and maintained baseline levels until the end of our sampling period. (Table 8).

**Table 5:** Differences between conjugated estrone concentrations in prepartum samples at different stages of gestation. Groups that differ from the reference group (late gestation) are indicated in bold (the credible interval does not overlap zero). Intercept: advanced gestation (between −60 days and parturition); beginning of gestation (days −347 and −240), early gestation (days −220 and −160), middle gestation (days −140 and −80), n_eff_ = effective number of Markovian chains

Stage of gestation	Mean	Credible intervals		n_eff_
		25%	95%	
**Intercept**	**983.7**	**769.6**	**1187.7**	**4000**
**Beginning of gestation**	**−935.7**	**−1253.6**	**−620.6**	**4000**
**Early gestation**	**−915.3**	**−1258.7**	**−598.8**	**4000**
**Middle gestation**	**−748.7**	**−1090.0**	**−438.6**	**4000**

**Table 6:** Differences in estrone conjugates concentrations between calving day and postpartum days. Groups that differ from the reference day (calving day) are indicated in bold (the credible interval does not overlap zero). Intercept: birth date, n_eff_ = effective number of Markovian chains

Postpartum days	Mean	Credible intervals		n_eff_
		25%	95%	
**Intercept**	**1719**	**1408**	**2014**	**4000**
**One**	**−1388**	**−1764**	**−1016**	**4000**
**Two**	**−1472**	**−1841**	**−1101**	**4000**
**Three**	**−1558**	**−1998**	**−1164**	**4000**
**Four**	**−1574**	**−1991**	**−1136**	**4000**
**Five**	**−1551**	**−2043**	**−1091**	**3795**
**Six**	**−1580**	**−1962**	**−1208**	**3821**

**Table 7:** Differences between PdG concentrations in prepartum samples at the different gestational stages analyzed. Groups that differ from the reference group (advanced gestation) are indicated in bold (the credible interval does not overlap zero). Intercept: advanced gestation (between −60 days and calving); beginning of gestation (days −347 and −240), early gestation (days −220 and −160), middle gestation (days −140 and −80), n_eff_ = effective number of Markovian chains

Stage of gestation	Mean	Credible intervals		n_eff_
		25%	95%	
Intercept	88.516	73.548	104.182	4900
Beginning of gestation	−22.269	−44.583	2.935	4997
**Early gestation**	**−25.164**	**−50.897**	**−2.511**	**4900**
**Middle gestation**	**−37.819**	**−63.682**	**−12.448**	**4900**

**Table 8:** Differences in pregnanediol concentrations between calving day and postpartum days. Days that differ from the reference day (calving day) are indicated in bold (the credible interval does not overlap zero). Intercept: birth date, n_eff_ = effective number of Markovian chains

Days postpartum	Media	Credible intervals		n_eff_
		25%	95%	
**Intercept**	**139.30**	**102.88**	**178.74**	**4000**
**One**	**−70.35**	**−120.11**	**−26.88**	**4837**
**Two**	**−92.43**	**−137.23**	**−41.82**	**4000**
**Three**	**−86.42**	**−139.03**	**−32.47**	**3855**
**Four**	**−88.18**	**−145.27**	**−38.75**	**4000**
**Five**	**−86.81**	**−148.30**	**−27.96**	**4000**
**Six**	**−62.04**	**−109.45**	**−10.59**	**4000**

### Extrapolation of experimental results to the wild population

The proportion of pregnant and non-pregnant females at the beginning and end of gestation was assessed in a wild, free-ranging guanaco population. In the study under captive conditions, all females resulted pregnant; therefore, retrospective hormonal data of non-pregnant females obtained in previous work by our research group was used (Marozzi *et al*., 2020). As stated before, estrogens remained basal during the first stages of gestation (Fig. 3); hence, only variations in progestogen metabolite concentrations were used to diagnose early pregnancy.

**Fig. 4:**
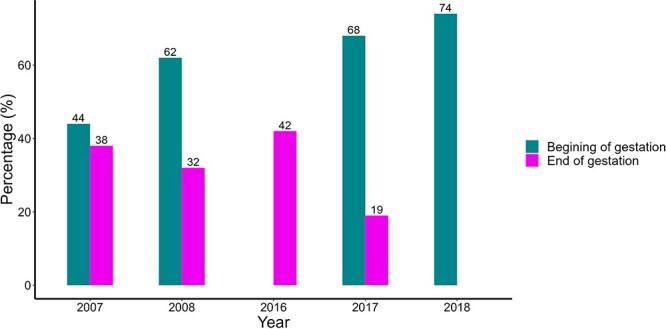
Percentage of pregnant females during the beginning and end of gestation in the guanaco population of La Payunia. Numbers above each column represent the percentage of pregnant females.

More than 50% of the females were pregnant during the summer season (early gestation, Fig. 4), except in 2007 in which the proportion of pregnant females was lower. Some samples could not be assigned to the pregnant/non-pregnant categories (4 in 2017 and 11 in 2018) because PdG concentrations did not fit into either of the two; thus, they were discarded.

To assign the percentages of pregnant and non-pregnant females in the spring season (advanced gestation), we combined the information obtained from EC and PdG concentrations (Fig. 4). At this stage, the proportion of pregnant females was lower than the proportion of non-pregnant females (<50% each year, Fig. 4). The proportion test indicated that the proportion of pregnant females was significantly different among years compared with non-pregnant females during early pregnancy and late pregnancy (early pregnancy: χ^2^ = 21.392, *P* = 0.00008727; late pregnancy: χ^2^ = 13.746, *P* = 0.003272), indicating how variable this parameter was among years in a wild population.

## Discussion

This is the first study that biologically validates a non-invasive method for monitoring hormonal fluctuations during gestation in guanacos. Our results demonstrate that the hormonal changes that support gestation can be appropriately assessed in guanaco feces. As expected, the concentration of progestagens increased gradually at the late stages of pregnancy and decreased sharply after calving. The secretory profile evaluated in blood samples reported by Riveros *et al*. (2009) indicated a gradual decrease in progesterone levels during the last 4 weeks of gestation. Due to the mechanisms involved in steroid hormone excretion, the results observed in feces have a 24- to 72-h delay compared to what occurs in the blood (Palme, 2005; Kersey and Dehnhard, 2014). As expected, in our study, PdG concentrations decreased on the first day post-calving. Estrogens increased in the last days before calving and declined after calving; as with progestagens, a delay was observed compared with the secretory profile in the blood (Fig. 3).

This type of study is of great importance since it sets precedents applicable to other wild populations. Studies investigating progestagens variations in ungulates include multiple species (e.g. *Moschus chrysogaster*; Mithileshwari *et al*., 2016, *Mazama gouazoubira*; Pereira *et al*., 2006, *Cervus elaphus*; White *et al*., 1995). In particular, Schwarzenberger *et al*. (1995) evaluated hormonal changes during the early stages of gestation in the vicuña (*Vicugna vicugna*), the other wild South American camelid, whose gestation cycle is similar to guanacos’ (Fowler and Bravo, 2010). The authors observed an increase in progestagens at the beginning of pregnancy that remained elevated until mid-pregnancy. The maximum concentrations of progestagens were observed in week 10 of gestation (Schwarzenberger *et al*., 1995). However, guanacos showed a marked increase in progestagens only after week 30 of gestation (~80 days before calving) and maintained these levels until the end of gestation. Schwarzenberger *et al’s* (1995) study ended several months before calving; therefore, the last stages of gestation cannot be compared, nor can estrogen concentrations, which the authors did not evaluate.

Since progestagens are the best predictors of pregnancy, the study of this hormone variation has generally received more attention from researchers than estrogen fluctuations (e.g. Kirkpatrick *et al*., 1993; Garrott *et al*., 1998; Schoenecker *et al*., 2004; Mithileshwari *et al*., 2016; Flacke *et al*., 2017). Estrogen excretory profile is usually more variable, so they are not considered good predictors of pregnancy (Lasley and Kirkpatrick, 1991; Hundertmark *et al*., 2000; Knott *et al*., 2013; Mastromonaco *et al*., 2015; Nagl *et al*., 2015). However, in guanacos, our results emphasize the fact that the information provided by EC concentrations has the potential to be a calving indicator, using a methodology that avoids animal handling when pregnancy is advanced, and more invasive treatments could put the fetus’s life at risk (Solberg *et al*., 2003). Thus, it would be advisable to include the information provided by estrogen fecal metabolites as well, to allow a more accurate pregnancy diagnosis in free-ranging wild animals. Therefore, for late pregnancy diagnosis, between 90 and 30 days prepartum, measuring PdG and EC fecal metabolites is adequate for a proper assessment.

Regarding the results obtained in wild conditions, depending on the stage of pregnancy, i.e. early or late, the timing of sampling in wild conditions is relevant. For early pregnancy diagnosis, it is advisable to sample females at 3–4 months of gestation (March or April), as physiological variability among individuals in the first 2 months hinders the correct assignment of pregnancy status. Previous research on guanacos indicates that hormone concentrations in non-pregnant females are markedly lower than in pregnant females (Riveros *et al*., 2009; Marozzi *et al*., 2020). Thus, if progestagens levels in samples collected during the austral fall are significantly higher than the expected range for non-pregnant females (>45.4 ± 24.4 μg/g; Marozzi *et al*., 2020), the female can be considered pregnant. The proportion of early and late pregnant females in wild conditions was significantly variable among years. Although they were collected randomly, the consistent observation of a lower proportion of pregnant females at the end of gestation suggests that there are instances where gestation does not reach full term. Possibly, such differences could be due to spontaneous abortions caused by hormonal or metabolic variations (Fowler and Bravo, 2010), parasitic infections (Kreizinger *et al*., 2015) or to other factors such as climate or primary productivity, which may influence gestation success (Gittleman and Thompson, 1988; Hamel *et al*., 2010). Furthermore, abortions may impact birth rate and recruitment and, consequently, the species conservation (Creel *et al*., 2007; Cotterill *et al*., 2018; Vitikainen *et al*., 2019). Given that climate change influences nutritional resource availability and parasitic infection prevalence, it is relevant to consider the effect of abortion on population parameters in future research (Root *et al*., 2003; Dimac-Stohl *et al*., 2018).

Females of wild ungulates tend to favor survival over reproduction (Hamel *et al*., 2010; Anouk Simard *et al*., 2014). After conception, pregnancy success will be mainly determined by environmental conditions, such as heavy snowfalls that reduce access to vegetation and the possibility of accumulating nutrients (Anouk Simard *et al*., 2014). Consequently, females in poor body condition may not be physiologically able to sustain pregnancy (Kirkpatrick *et al*., 1993; Albon *et al*., 2017). Future research should focus on understanding the influence of nutrient availability and females’ nutritional state during the gestational process (Russell *et al*., 1998). Wild ungulates are an extraordinarily diverse group of mammals with substantial variation in their reproductive biology, whether in their anatomy, behavior or seasonality (Sontakke, 2018). Therefore, acquiring basic knowledge of their reproduction poses a significant challenge to address before making management decisions that may influence this sensitive aspect of their life.

## Data Availability

Data is available on reasonable request.
